# Epicardium-myocardium crosstalk orchestrates heart development

**DOI:** 10.3389/fcell.2025.1655878

**Published:** 2025-09-24

**Authors:** Anika Nusrat, Mingfu Wu

**Affiliations:** Pharmacological and Pharmaceutical Sciences, College of Pharmacy, University of Houston, Houston, TX, United States

**Keywords:** epicardium, myocardium, crosstalk, morphogenesis, development

## Abstract

The epicardium is critical in heart development, functioning as a paracrine signaling hub and a source of progenitor cells. Bidirectional communication between the epicardium and myocardium, mediated by tightly regulated signaling networks, is essential for proper cardiac morphogenesis. This review presents a comprehensive overview of epicardium-myocardium crosstalk across species, emphasizing how this crosstalk influences epicardial epithelial-to-mesenchymal transition (EMT), fate specification of epicardium-derived cells (EPDCs), myocardial proliferation and growth, and coronary vasculature development. We critically assess decades of research elucidating key pathways—retinoic acid, fibroblast growth factor (Fgf), insulin-like growth factor (Igf), platelet-derived growth factor (Pdgf), transforming growth factor-β (Tgfβ), various transcriptional and epigenetic regulators, as well as calcium signaling mediated epicardial function—that coordinate these developmental processes. Additionally, we include detailed tables summarizing key experimental models and mechanistic insights that have shaped the field. This integrative analysis advances our current understanding of epicardial-myocardial crosstalk and highlights unresolved questions to guide future investigations into cardiac development and disease.

## 1 Introduction

The epicardium, the outermost layer of the heart, has emerged as a key orchestrator of cardiac morphogenesis and repair (Kurkiewicz, 1909; Pennisi et al., 2003; Hiruma and Hirakow, 1989; Quijada et al., 2020). During embryogenesis, mesothelial cells from the proepicardial organ (PEO) migrate to the heart surface, forming a continuous epithelial sheet that constitutes the early epicardium (Cao et al., 2020). As development progresses, a subset of epicardial cells undergoes epithelial-to-mesenchymal transition (EMT), delaminating from the surface and differentiating into epicardium-derived cells (EPDCs) (Wu et al., 2010; Wessels and Pérez‐Pomares, 2004). These EPDCs contribute to various cardiac lineages within the heart, including fibroblasts, vascular smooth muscle cells, and pericytes (Gittenberger-de Groot et al., 1998; Dettman et al., 1998). Thus, the epicardium not only provides cellular input to the developing heart but also serves as a signaling nexus, coordinating myocardial growth and coronary vessel formation through paracrine factors.

Communication between the epicardium and myocardium occurs via finely tuned paracrine signaling pathways, including those involving retinoic acid (RA) (Merki et al., 2005; Brade et al., 2011), fibroblast growth factors (Fgfs) (Pennisi and Mikawa, 2009; Lavine et al., 2005; Torlopp et al., 2010; Vega-Hernández et al., 2011), insulin-like growth factors (Igfs) (Li et al., 2011; Shen et al., 2015), platelet-derived growth factors (Pdgfs) (Kang et al., 2008; Mellgren et al., 2008), and transforming growth factor-β (Tgfβ) (Austin et al., 2008; Sánchez and Barnett, 2012; Clark et al., 2016). These pathways govern various aspects of myocardial development, while epicardium-intrinsic signals (Wu et al., 2013) and extracellular matrix remodeling (Craig et al., 2010a; Allison et al., 2015) further influence this dynamic interplay. Importantly, epicardial cells also respond to myocardial cues, emphasizing the bi-directional nature of this communication, critical for heart development.

Recent advances have deepened our understanding of epicardial biology, particularly in lineage tracing, cellular heterogeneity, transcriptional regulation, noncoding RNAs, and chromatin remodeling (Lupu et al., 2020; Villa del Campo et al., 2016; Tyser et al., 2021; Harvey et al., 2024; Jang et al., 2022). Investigations using avian, zebrafish, and mammalian models, along with human pluripotent stem cell-derived epicardial cells, have provided valuable platforms for elucidating the roles of the epicardium in heart development (Meier et al., 2023; Männer et al., 2005; Kim et al., 2010).

This review comprehensively synthesizes past and current knowledge of epicardial-myocardial interplay that has shaped our understanding of how the epicardium communicates with myocardium to regulate heart morphogenesis. By systematically organizing and critically evaluating the chronological progression of discoveries, we highlight key regulatory mechanisms and delineate outstanding questions to further drive research in this evolving field.

## 2 Development of proepicardium, epicardium, and myocardium

At the very beginning of heart development (around embryonic day E7.5 in mouse (Li et al., 2011), 4 weeks of gestation in human (Cao and Cao, 2018)), the heart is composed of inner endothelium and outer myocardium. Around E9.5–E10.5 in mice, a third layer, the epicardium, starts to cover the naked surface of the myocardium. Precursor cells for the epicardium originate from a structure called PEO, which comprises a sheet of mesothelial cells and is located at the venous pole of the heart (Cao et al., 2020). Mesothelial cells from PEO can migrate to the heart surface by four mechanisms. First, a cluster of mesothelial cells form cyst-like structures in mice and travel through the pericardial cavity towards the myocardium (Sengbusch et al., 2002; Li J. et al., 2017; Komiyama et al., 1987). Second, the mesothelial bridge is formed between PEO and myocardium to enable mesothelial cells to travel and attach to the myocardial surface, as reported in chick (Nahirney et al., 2003), *xenopus* (Jahr et al., 2008), and zebrafish (Plavicki et al., 2014). A connecting bridge made of extracellular matrix components was reported to carry mesothelial cells to the chick myocardium (Nahirney et al., 2003). Third, free-floating mesothelial cells come in direct contact and attach to the myocardium in mice (Li J. et al., 2017; Rodgers et al., 2008). Heartbeat-mediated fluid forces from blood flow and the pericardial fluid can drive this migration through the pericardial cavity (Peralta et al., 2013). Fourth, villi-like structures protrude from the PEO and carry the mesothelial cells near to the myocardium (Li J. et al., 2017; Rodgers et al., 2008). Notably, these migratory modes are not necessarily exclusive, as multiple mechanisms can be adopted simultaneously (Li J. et al., 2017). After establishment of the epicardial layer, a unique set of transcriptional and surface markers defines its identity and regulates its function (Moore et al., 1999; Kraus et al., 2001; Mahtab et al., 2008; Pryce et al., 2007). At around E10.5 in mice, paralleling the epicardium development, thin-layered myocardium starts to expand via cell proliferation and migration (Li et al., 2016) and achieves two distinct zones: the trabecular zone facing inward, and the compact zone close to the epicardium. The development of myocardium has an intrinsic relationship with epicardium (Li et al., 2011), which is the major focus of this review.

## 3 Epicardial fate mapping and fate switch

Epicardial cells contribute to several lineages of the heart by generating EPDCs via EMT. EMT is a process conserved across species, which starts at chick at HH (Hamburger-Hamilton) stage 16, E12.5 in mice, and fetal stage 3 in humans (Quijada et al., 2020; Risebro et al., 2015). During EMT, epicardial cells undergo perpendicular division or directional migration (Wu et al., 2010) to enable cells to enter the myocardium. At this early stage, epicardial cells are not typical epithelial cells, and disrupting their polarity and cell-cell adhesion (marked by downregulation of epithelial and adhesion markers: cytokeratin (Kim et al., 2010), E-cadherin (von Gise and Pu, 2012), ZO-1 (Compton et al., 2006), β-catenin (Merki et al., 2005)) promotes their mesenchymal migratory properties (marked by upregulation of N-cadherin (Sridurongrit et al., 2008), Vimentin, Snai1, Twist1). Following EMT, EPDCs invade the subepicardial space and myocardium to give rise to multiple cardiac lineages.

However, no single marker can identify exclusively all epicardial cells and EPDCs because of their complexity and heterogeneity. Wt1 (Moore et al., 1999), Tbx18 (Kraus et al., 2001), Raldh2 (Du et al., 2023), Podoplanin (Mahtab et al., 2008), Scx (Pryce et al., 2007), Sema3D (Katz et al., 2012), Tcf21 (Lupu et al., 2020) have been widely used as classical markers to define epicardial cells and derivatives. Additionally, several other markers have been delineated from time to time by several research groups to label epicardial cells and EPDCs (Lupu et al., 2020; Mahtab et al., 2008; Du et al., 2023; Moss et al., 1998; Perez-Pomares et al., 2002; Smith et al., 2011). All the markers have specific context and time-dependent expression patterns. A summary of the epicardial markers (including gold standards as well as experimentally utilized markers) is discussed in Table 1.

**TABLE 1:** List of epicardial cell markers during heart morphogenesis.

Marker	Type/localization	Cardiac tissue specific expression	Species
Wt1	Zinc finger transcription factor (nuclear expression)	PEO, early epicardium, subepicardial mesenchyme, EPDCs (Moore et al., 1999), endothelial cells (Lupu et al., 2020; Rudat and Kispert, 2012), cardiomyocytes (Zhou et al., 2008; Duim et al., 2016)	Mouse, chick, zebrafish
Tbx18	T box-Transcription factor (nuclear expression)	PEO, epicardium (Kraus et al., 2001), EPDCs (fibroblast, coronary smooth muscle cells), cardiomyocytes in Interventricular septum, atrial and ventricular wall (Cai et al., 2008)	Mouse, chick, zebrafish
Tcf21 (Capsulin/Pod1/Epicardin)	bHLH Transcription factor	Proepicardium, Epicardium, Post-EMT epicardium-derived fibroblast specific expression (Acharya et al., 2012; Braitsch et al., 2012), Resident and activated fibroblast in adult heart (Kanisicak et al., 2016)	Mouse, zebrafish, *Xenopus* (Tandon et al., 2013)
Sema3D	Secreted protein	Epicardium, Proepicardium, EPDCs (Lupu et al., 2020), early sinus venosus, endocardium (Katz et al., 2012)	Mouse, Chick, zebrafish
Upk1b and Upk3b	Transmembrane protein	Embryonic and Matured epicardium (Du et al., 2023; Knight-Schrijver et al., 2022)	Mouse
Podoplanin	Transmembrane glycoprotein	Epicardium, few EPDCs (Mahtab et al., 2008)	Mouse
Caveolin-1	Pan epicardial marker, membrane protein	Adult epicardium (injured and uninjured, zebrafish) (Cao et al., 2016)	Mouse
Raldh2	Enzyme, cytoplasmic, epicardial surface	Epicardium (Moss et al., 1998; Perez-Pomares et al., 2002)	Mouse, chick
Cytokeratin	Intermediate filament protein	Epicardial epithelial cells (Peeters et al., 1995; Landerholm et al., 1999)	More common in chick
Itga4 (α4-integrin)	Cell surface receptor	Proepicardium, Epicardium, endocardial cushion, outflow tract (Yang et al., 1995; Dettman et al., 2003; Palmquist-Gomes et al., 2023)	Mouse, Chick (Dettman et al., 1998)
Gata4	Transcription factor (Nuclear)	Proepicardium, cardiomyocyte, smooth muscle cell, endothelial cells (Zhou et al., 2008)	Mouse
Snai1 (Snail)	Zinc Finger Transcription factor	EMT marker in epicardium (Martínez-Estrada et al., 2010), in endocardium (Niessen et al., 2008)	Mouse
Pdgfrα	Receptor tyrosine kinase	Epicardium, Epicardium-derived fibroblast (Smith et al., 2011), Endocardium derived fibroblast (Ali et al., 2014), cardiomyocytes (specially in adult injured heart (Chong et al., 2013)	Mouse
Pdgfrβ	Receptor tyrosine kinase	EPDCs (smooth muscle progenitors) (Smith et al., 2011), coronary vascular and plexus (Mellgren et al., 2008)	Mouse, Chick
Scleraxis (Scx)	Transcription factor	Proepicardium, Epicardium, low expression in EPDCs (Lupu et al., 2020), early sinus venosus, endocardium (Katz et al., 2012)	Mouse
Slug/Snai2	Zinc Finger Transcription factor	EMT marker in epicardium (Martínez-Estrada et al., 2010), Endocardial cushion (Niessen et al., 2008)	Mouse
Twist1	bHLH Transcription factor	Broad EMT regulator (von Gise and Pu, 2012; Zhou et al., 2010)	Mice, Chick
Gata5 (Merki et al., 2005)	Transcription factor	Proepicardium and epicardium, septum transversum (ST)	Mice, Chick (Kolander et al., 2014), Zebrafish (Reiter et al., 1999), *Xenopus* (Haworth et al., 2008)
Sox9	Sry-related transcription factor	Epicardial EMT/mesenchymal EPDC marker (Quijada et al., 2020; Smith et al., 2011)	Mouse
Zeb1/2	Transcription factor	Epicardium, Valve, and cushion mesenchyme (Debnath et al., 2022)	Mouse
Mesothelin (Msln)	Membrane glycoprotein	Matured epicardium, enriched in ECM signaling (Du et al., 2023)	Mouse
Efemp1/Fibulin-3	ECM/Secreted protein	Matured epicardium, enriched in ECM components (Du et al., 2023)	Mouse
C3	Secreted protein	Matured epicardium, enriched in ECM signaling (Du et al., 2023)	Mouse

Tracing of EPDCs has been intensively investigated for years and is still ongoing across chick, mice, zebrafish, *xenopus*, and humans. At the beginning, dye or retroviral vectors were employed to label epicardial cells and EPDCs in chick embryos (Peeters et al., 1995; Gittenberger-de Groot et al., 2000; Morabito et al., 2001). Eventually, genetic fate mapping tools were employed, such as epicardial promoter-driven *Cre* recombinase-mediated recombination to express β-galactosidase or fluorescent reporters, mostly in mouse models. The standard epicardial constitutive *Cre* lines, as well as the inducible lineages (*CreERT2*), are discussed in Table 2, detailing their spatiotemporal expression.

**TABLE 2:** List of standard epicardial lineages that label epicardial cells/EPDCs during development.

Cre line	Driver type	Fate‐Mapped cell types
*Wt1* ^*Cre/+*^ (Zhou et al., 2008)	Knock In	Proepicardium, Epicardium, EPDC (smooth muscle cell) (Zhou et al., 2008), Endothelial cells (Rudat and Kispert, 2012; Zhou et al., 2008; Zhou and Pu, 2012), Cardiomyocyte (present in all chambers) (Rudat and Kispert, 2012; Zhou et al., 2008; Zhou and Pu, 2012), fibroblast of annulus fibrosis (Zhou et al., 2010), adipose tissue (Simões and Riley, 2018)
*mWt1/IRES/GFP-Cre (Wt1Cre)* (del Monte et al., 2011; Wessels et al., 2012)	Transgenic	EPDC-derived fibroblast in walls, Valve interstitial cells (Wessels et al., 2012)
*Wt1* ^*CreERT2/+*^ (Zhou et al., 2008)	Knockin	Mesoderm including cardiomyocyte before E9.5 (Zhou et al., 2008), epicardium at E11.5 (Zhou et al., 2008), Endothelial cells after E12.5 (Rudat and Kispert, 2012; Zhou and Pu, 2012; Tallquist, 2020)
*Tbx18* ^*Cre/+*^ (Cai et al., 2008)	knock-in	Proepicardium epicardium, EPDC, cardiomyocyte in interventricular septum, atrium, and ventricle (Cai et al., 2008; Christoffels et al., 2009), adipose tissue (Simões and Riley, 2018)
*Tbx18* ^*CreERT2/+*^ (Guimarães-Camboa et al., 2017)	knock-in	Predominantly vascular mural cells (Guimarães-Camboa et al., 2017; Liu et al., 2024), fibroblast, smooth muscle cells, pericytes, SA node, and venous myocardium (induction at adult) (Guimarães-Camboa et al., 2017)
*Tcf21* ^*MerCreMer/+*^ (Acharya et al., 2011)	knock-in	EPDCs → fibroblasts, SMCs (Acharya et al., 2012); Fibroblast in adult (Kanisicak et al., 2016)
*Sema3D* ^*-Cre/+*^ (Katz et al., 2012)	knock-in	PE, Epicardium, SMCs, valves, endothelial, and cardiomyocyte (Katz et al., 2012)
*Scx* ^*Cre/+*^	knock-in	Early PE/epicardium, SMC, fibroblasts, endothelial cells, and minor CM (Katz et al., 2012), valves (Levay et al., 2008)
*Gata5Cre* (Merki et al., 2005)	transgenic	Early PE/epicardium, fibroblasts, SMCs, Septum transversum (Merki et al., 2005)
*Msln* ^*CreERT2/+*^ (*Mesothelin*) (Liu et al., 2014)	knock-in	embryonic/adult (Liu et al., 2014), Mesothelium-derived cells, fibroblasts, SMCs; no CM/EC contribution
*Ck19* ^*CreERT2/+*^ (Sekiya and Suzuki, 2012)	knock-in	Epicardial mesothelium, some endoderm (E10.5 onward)

Decades of epicardial research provide a strong consensus that EPDCs contribute extensively to cardiac fibroblasts, coronary vascular smooth muscle cells (cVSMCs), and pericytes during heart development (Zhou et al., 2008; Acharya et al., 2012; Braitsch et al., 2012; Fang et al., 2016). Lineage tracing via *Cre* recombinase driven by *Wt1*, *Tbx18*, and *Tcf21* locus consistently labels fibroblast and smooth muscle cells (SMCs) lineages during normal heart development, confirming that these are the major fates for EPDCs (Zhou et al., 2008; Acharya et al., 2012; Braitsch et al., 2012; Fang et al., 2016). Also, *Wt1* ^*cre/+*^ and *Tbx18* ^*cre/+*^ lineage-mediated EDPCs’ contribution to adipose tissue has been consistently reported (Yamaguchi et al., 2015; Chau et al., 2014).

However, a long-standing controversy sustains whether EPDCs can also contribute to cardiomyocytes and endothelial cell population of the heart. Several early studies detected cardiomyocyte labeling using constitutive *Wt1* ^*cre/+*^ and *Tbx18* ^*cre/+*^ lineages, implying that epicardial cells may contribute to myocardial lineage (Zhou et al., 2008; Cai et al., 2008; Zhou and Pu, 2012). If so, it raises another question: whether epicardial marker expression detected in cardiomyocytes conveys myogenic potential of epicardial differentiation, or instead, indicates early co-expression in common progenitor pools.

Villa del Campo et al. (2016) addressed these questions and showed that constitutive *Wt1-Cre* recombination can label a minor cardiomyocyte population—likely from early *Wt1+* progenitors prior to epicardial adhesion to the ventricle (extra cardiac). Moreover, inducible *Wt1* ^*creERT2/+*^ failed to mark cardiomyocytes, suggesting that true epicardium-derived myogenesis is minimal or absent once the epicardium is established. Similarly, Tyser et al. (2021) identified a juxta-cardiac field (JCF) — the earliest known mesoderm progenitor population contributing to both cardiomyocytes and proepicardial cells in mice. This might explain a partial overlap of *Wt1*/*Tbx18* expression with cardiomyocytes in constitutively expressed lineages. A previous finding by van Wijk et al. (2009) also supported the concept that epicardium and myocardium share a common progenitor pool. Additionally, using *Tcf21* ^*CreERT2/+*^ labeled lineage, Kikuchi et al. (2011) and Acharya et al. (2012) failed to detect epicardial *Tcf21+* cells’ contribution to cardiomyocytes and endothelial cells in zebrafish and mouse models, respectively.

In the quest for epicardial differentiation to coronary endothelial cells, early avian studies observed PEO-derived endothelium (Perez-Pomares et al., 2002; Guadix et al., 2006), while murine studies indicated minimal (Rudat and Kispert, 2012) or no such contribution (Zhou et al., 2008; Cai et al., 2008). Katz et al. (2012) resolved this conflict by revealing that PEO is a molecularly compartmentalized structure in mice, where specific subpopulations contribute to different cardiac lineages. For example, *Scx+* and *Sema3D +* PEO subpopulations contribute to the sinus venosus and endocardium of the coronary endothelium (Smits et al., 2018).

Whether epicardial fate commitment occurs before or after EMT has also been a central question over the years. Acharya et al. (2012), using *Tcf21* ^*iCre/+*^ lineage mapping and *Tcf21* null embryos, proved that *Tcf21+* epicardial cells specification precedes the onset of EMT, denying the previous assumption that EPDCs fate is not specified until EPDCs enter the myocardium (Dettman et al., 1998). Consistently, Miao et al. (2021) revealed that the fate of smooth muscle cells is pre-specified in PEO. However, a recent report by Lupu et al. (2020) showed broad co-expression of Wt1, Tbx18, Tcf21, Sema3d, and Scx in the PEO up to E13.5 stage, suggesting that exclusive fate specification may not occur until after EMT. Altogether, these findings imply heterogeneity in the timing of epicardial specification commitment across sub-lineages.

### 3.1 Epicardial fate switch/lineage transition

Epicardium has multipotent plasticity and can be sensitive to extrinsic signaling cues during its lineage specification. Recent studies have refined our understanding of how transcription factors and environmental cues coordinate this fate switch. A proper balance of epicardial signaling is indispensable to ensure correct lineage specification. For example, *Tcf21* null embryos manifested the absence of fibroblast differentiation, but SMC differentiation was not affected (Acharya et al., 2012). Interestingly, temporal loss of *Tcf21* expression in epicardial cells switched the fate specification from fibroblast towards cVSMC (Braitsch et al., 2012), implying that Tcf21 must repress transition to SMC and maintain EPDCs in a fibroblast state. Recently, Xiao et al. (2018) demonstrated that deletion of Hippo kinases *Lats1/2* in the mouse epicardium caused EPDCs to remain in a transitional state, leading to marked disruption in fibroblast differentiation.

Extrinsic signaling can induce epicardial plasticity toward myocardial fate. mYc (regulator of cell growth and proliferation) overexpression led to expansion of *Wt1* ^*cre/+*^ expression into cardiomyocyte (Villa del Campo et al., 2016). According to Kruithof et al. (2006), chick PE/ST *ex vivo* culture causes spontaneous differentiation into beating cardiomyocytes. This cardiomyogenic bias was enhanced by Bone Morphogenetic Protein (Bmp) and inhibited by Fgf signaling. Recently, Dueñas et al. (2020) revealed several non-coding RNAs, such as miR-195 in association with miR-125, miR-146, and miR-223, to regulate Fgf/Bmp-mediated cardiogenic potential of PEO in chicken. Consistently, a previous study by van Wijk et al. (2009) revealed that Bmp-Smad signaling shifts the lineage into the myocardium, while Fgf-Mek1 signaling causes a fate shift to epicardium. Thus, a misbalance between Fgf and Bmp might cause the developmental shift from epicardium into myocardium. However, these findings may not generalize across species, as Garcia-Padilla et al. (2022) could not replicate this cardiomyogenic shift in mouse PE explants, indicating interspecies differences.

Interestingly, the fate switch can be bidirectional. According to Marques et al. (2022), ectopic expression of Wt1a/b in zebrafish cardiomyocytes induces their trans-differentiation into epicardial-like cells. This suggests that in certain contexts, Wt1 reactivation can destabilize cardiomyocyte fate and trigger epicardial gene programs.

All together, these findings indicate that epicardial fate decisions remain highly plastic and context dependent. Balanced transcriptional, epigenetic, and extracellular signaling cues are essential for forming fibroblasts and coronary vasculature. Also, epicardial lineage specification largely excludes myocardial or endothelial fates under normal physiological conditions during development.

## 4 Epicardium-myocardium crosstalk

Epicardium plays versatile roles in heart development by exerting both non-cell-autonomous and cell-autonomous influences on myocardium. By regulating EMT and multifaceted paracrine signaling, providing EPDCs, ECM regulation, and guiding coronary vasculature formation, the epicardium tightly controls critical aspects of heart development, such as cardiomyocyte proliferation and maturation, ventricular wall compaction, and vascular system formation (Merki et al., 2005; Li et al., 2011; Zamora et al., 2007). Diverse experimental platforms, including mechanical interference, quail–chick chimeras, epicardial explant, organ culture, and more recently, conditional genetic mouse models have guided us to decipher these critical roles over decades. This section extensively reviews the evolution of the findings and our understanding of indispensable epicardial–myocardial communication during cardiac morphogenesis.

### 4.1 Non-autonomous epicardial regulation of myocardium

#### 4.1.1 1993–2000 | Foundational models defining epicardial necessity in heart development

The pioneer understanding of epicardial significance in heart development emerged with a novel ‘quail-chick chimera’ technique, where quail PEO was grafted into the host chick embryonic heart (Poelmann et al., 1993). This innovation led to myriads of epicardium-focused research in the following years. Utilizing this technique, Gittenberger-de Groot et al. (1998) first demonstrated that EPDCs populate the myocardium, sub-endocardium, and atrioventricular cushion during heart development. In a parallel study, Dettman et al. (1998) established a primary epicardial explant culture system and specified EPDCs as cVSMCs, perivascular and intermyocardial fibroblasts (Zhou et al., 2008; Vrancken Peeters et al., 1999). First evidence of direct epicardial regulation of heart development was revealed when a complete or partial PEO inhibition in chick embryos (Männer, 1999) led to several myocardial defects, i.e., impaired coronary formation, interventricular septation, and myocardium expansion (Gittenberger-de Groot et al., 2000). In those days, implantation of eggshell membrane (Pennisi et al., 2003; Gittenberger-de Groot et al., 2000; Männer, 1993; Poelmann et al., 2002) and mechanical excision of PEO villi (Perez-Pomares et al., 2002) were the microsurgical techniques used to generate PEO/epicardial block. However, these approaches either caused incomplete inhibition or just a delay in the migration of PEO-derived cells. Later, Männer et al. (2005) introduced a photoablation strategy that enabled permanent epicardium blockade.

##### 4.1.1.1 Adhesion molecules and other mediators

Meanwhile, epicardial necessity in heart development was testified with the advent of genetic models, such as deletion of *Vcam-1* (vascular cell adhesion molecule 1 (Kwee et al., 1995)) and *α4-integrin* (cell surface receptor protein mediating ECM and cell-cell adhesion (Yang et al., 1995)) in mice (Pérez-Pomares and de la Pompa, 2011). *Vcam-1* null mice exhibited a complete absence of epicardial formation, a thinned myocardium, and septal defects. Similar defects were observed in *α4-integrin* deficient embryos as well. Phenotypic manifestation of *Vcam-1* and *α4-integrin* knockout (KO) closely mirrored that of *Wt1* null mutants (absence of a definitive epicardial layer, thinning of myocardial wall) (Kreidberg et al., 1993). Wt1 (Wilms Tumor 1) is a zinc finger transcription factor, which by this time, was already detected in the proepicardium, epicardium and subepicardial mesenchyme of mice and chick heart (Moore et al., 1999). Hence, these phenotypic findings implied epicardium-mediated defects in all three models.

In a follow-up study, Sengbusch et al. (2002) outlined that the migration of epicardial progenitors to the heart surface and maintenance of the already formed epicardium both require α4-integrin. Further, Dettman et al. (2003) applied the adenoviral system to delete *α4-integrin* and revealed that α4-integrin downregulation causes accelerated EMT, leading to premature invasion into the myocardium. Thus, α4-integrin was the first reported regulator to restrain EMT rather than promote it. Wu et al. (1999) discovered another regulator, Erythropoietin (Epo), to mediate myocardial growth. Mice lacking *Epo* and Epo receptor (*EpoR*) showed ventricular hypoplasia with myocyte proliferation and vasculature defects. Notably, significant Epo expression was observed in epicardium and endocardium, but not in cardiomyocytes, implying non-autonomous Epo mediated regulation of myocardium.

#### 4.1.2 2001–2015 | dissecting epicardial regulation: genetic models, paracrine signaling, and transcription factors

##### 4.1.2.1 Retinoic acid (RA) signaling

Based on the implication by earlier studies that the epicardium influences myocardium development, researchers started to dissect the underlying mechanisms. Chen et al. (2002) delineated early evidence of epicardial paracrine regulation of myocardium. They showed that epicardial cells secrete retinoic acid (RA) inducible mitogens to regulate cardiomyocyte cell cycle and proliferation. *RA receptor X* (*RXRα*)-deficient epicardial cells do not secrete these mitogens. Next year, Stuckmann et al. (2003), using the chick heart slice culture system, specified that epicardial RA (Chen et al., 2002) and Epo (Wu et al., 1999) signaling regulate myocardium growth via these mitogens, rather than directly stimulating cardiomyocytes. Consistently, Perez-Perez-Pomares et al. (2002) pointed out that ablation of chick PEO impaired Wt1 and Raldh2 (enzyme for RA synthesis) expression in EPDCs, leading to a thinned myocardium and defective vasculature.

Earlier, the global *RXRα* KO mouse model revealed myocardial thinning and midgestation lethality (Gruber et al., 1996), while myocardium-specific deletion produced no cardiac phenotype (Chen et al., 1998), implying that the myocardial defect is not cell-autonomous. Later, Merki et al. (2005) used the *Cre* recombinase system and generated an epicardial-specific transgenic mouse line *Gata5Cre* to delete *RXRα*, and these mutants mimicked the global *RXRα* KO phenotype. Mechanistically, reduced expression of Wnt9b, Fgf2, and β-catenin was revealed, indicating an epicardial RA–Wnt–Fgf signaling axis mediated non-autonomous regulation of myocardium. Additionally, Guadix et al. (2011) observed a significant reduction in Raldh2 expression in the epicardium of *Wt1* null mice, thus revealing Wt1 as another regulator of RA synthesis.

In a parallel study, Brade et al. (2011) uncovered a novel extracardiac-to-epicardial-to-myocardial signaling array. Authors identified that hepatic RA signaling induces hepatic expression of Epo, essential to induce Insulin growth factor (Igf2) mitogen in the epicardium. In the absence of RA or its receptor (*Raldh2* ^*−/−*^ (Lin et al., 2010) and *Rxrα* ^*−/−*^ mutants), hepatic Epo fails to induce epicardial Igf2, causing insufficient stimulation of myocardial proliferation and myocardial thinning. Notably, they revealed intact epicardial RA signaling in *Rxrα* ^*−/−*^ mutants. This suggests that the compact zone defect reported in *Rxrα* ^*−/−*^ hearts (Merki et al., 2005; Chen et al., 2002) might arise not from intrinsic epicardial RA loss, but possibly from a non-cardiac RA-dependent mechanism. However, previous reports of myocardial thinning in epicardium-specific *Rxrα* ^−/−^ mutants (Merki et al., 2005) indicate that epicardial Rxrα at least partially, if not all, contributes to the defects of global *Rxrα* ^−/−^ mutants.

##### 4.1.2.2 Igf (insulin growth factor) signaling

Paralleling the identification of Igf2 as epicardial mitogen (Brade et al., 2011), Li et al. (2011) uncovered that epicardial Igf2 is indispensable for myocardial proliferation as it cannot be compensated by endocardial sources. Shen et al. (2015) re-established the epicardial origin as well as sufficiency of epicardial Igf2 for compact zone growth using multiple genetic models. Conditional deletion of *Igf2* with *Tbx18* ^*Cre/+*^ (epicardial-specific) or *Nkx2.5* ^*Cre/+*^ (cardiac mesoderm-wide) resulted in a significant reduction of ventricular wall thickness. In contrast, deletion with *Tie2Cre* (endothelial/endocardial) or *Myh6Cre* (myocardial) had no such effect. They further focused on epicardial-myocardial Igf communication by pinpointing that global *Igf2* KO and myocardium-specific deletion of Igf receptors, Igf1r, and Insr, both models manifested ventricular hypoplasia associated with proliferation defects.

Later, Yan et al. (2018) demonstrated that, in addition to myocardial expression (Shen et al., 2015), *Igfr1* is also expressed in epicardium and contributes to myocardial growth via the Focal adhesion kinase (FAK) pathway. A recent study by Wang et al. (2019) specified that, between Igfr1 and Insr, Igfr1 predominantly binds with Igf2 while Insr is unable to compensate for the function of Igfr1. Findings in zebrafish model by Huang et al. (2013) further complement these studies, indicating similarity across species. Most recently, Meier et al. (2023) confirmed epicardial Igf2 and myocardial Igfr1 expression in a human “epicardioids” (a 3D human stem cell-derived organoids) and mirrored the necessity of mitogenic role of Igf2 in human fetal heart development. Igfr1 inhibition impaired myocardial proliferation and compaction as previously reported in mice (Yan et al., 2018), and recombinant Igf2 rescued these defects in organoids even in the absence of epicardium (in RA-deficient spheroid that lacks epicardium).

In addition to the extracardiac regulation of epicardial Igf2 revealed in earlier studies (Brade et al., 2011), Shen et al. (2015) further elucidated a biphasic regulation. While hepatic Epo regulates epicardial Igf2 at the earlier E10–11.5 stage, a placenta-dependent regulation takes over this process at E11.5 onward, both controlled by RA signaling. Placental regulation is further driven by optimal glucose uptake and normoxic conditions. Recently, Jang et al. (2022) added another dimension by introducing epigenetic regulation of epicardium to influence myocardium growth. Deletion of *Histone deacetylase* (*Hdac3*) in epicardium caused microRNA-mediated suppression of mitogens Fgf9 and Igf2, resulting in myocardial proliferation and compaction defects.

##### 4.1.2.3 Fgf (fibroblast growth factor) signaling

Mikawa (1995) and Mima et al. (1995) were among the first groups to introduce Fibroblast Growth Factor (Fgf) signaling in heart development. They revealed that myocardium-targeted Fgf receptor inhibition by antisense RNA led to a significant but transient defect in cell proliferation. Pennisi et al. (2003) first discovered non-autonomous regulation of Fgf by observing that thinner myocardium in epicardium-blocked chick hearts was caused by disrupted Fgf signaling components. They characterized that the epicardium is essential to maintain the correct level of Fgf mitogens required for myocyte proliferation, but the epicardium is not required for establishing the transmural pattern of mitogen expression or myocyte proliferation.

In mammals, Lavine et al. (2005) specified Fgf ligands Fgf9, Fgf16, and Fgf20 as epicardial- and endocardial-derived mitogens, while receptors Fgfr1 and Fgfr2c were found in the myocardium. *Fgf9* ^*−/−*^ mice showed reduced cardiomyocyte proliferation and ventricular hypoplasia. Notably, myocardium-specific *Fgfr1/2 KO* showed more severe hypoplasia than *Fgf9* ^*−/−*^ mice, indicating possible contributions from additional Fgf ligands (Li et al., 2024). Indeed, both Fgf16 (Hotta et al., 2008) and Fgf20 promoted proliferation in a Fgfr1/2-dependent manner (Lavine et al., 2005).

Similarly, ligands such as Fgf1, Fgf2, Fgf7, Vegf, and Egf were reported to enhance EMT in epicardial explant (Dettman et al., 1998) and avian heart culture system (Morabito et al., 2001). Further work by Torlopp et al. (2010) identified expressions of Fgf2, Fgf10, and Fgf12, and their receptors Fgfr1, Fgfr2, and Fgfr4 in chick PEO, supporting findings in quail (Pennisi and Mikawa, 2009). In their study, exogenous Fgf2 enhanced PE growth and EMT via Mapk and PI3K/Akt pathways, supporting findings by Morabito et al. (2001). Similar to Fgf ligands (Morabito et al., 2001), *Fgfr1* overexpression also enhanced epicardial EMT and myocardial invasion, and its knockdown significantly impaired the invasion of PE progenies (Pennisi and Mikawa, 2009). Notably, Fgf inhibition did not affect expression of proepicardial markers Tbx18, Wt1, and Tbx5, indicating that Fgf signaling may not regulate lineage identity (Pennisi and Mikawa, 2009).

Vega-Hernández et al. (2011) introduced bidirectional Fgf-mediated epicardial-myocardial communication in heart development. Authors revealed that myocardial Fgf10 binds with epicardial Fgfr1 and Fgfr2b to promote EPDC invasion into the myocardium. Interestingly, the deficiency in epicardium-derived cardiac fibroblasts was correlated with reduced cardiomyocyte proliferation and thin myocardium observed in *Fgf10* ^*−/−*^, *Fgfr2b−/−*, and *Wt1* ^*Cre/+*^ *; Fgfr1/2* mutants. It indicates that not only epicardial mitogen-driven signaling, but also the presence and function of epicardium-derived fibroblasts, are crucial for myocardial growth. However, conflicting findings were revealed by Rudat et al. (2013), who observed no defect in heart development in *Tbx18* ^*Cre/+*^ mediated loss of function of *Fgfr1/Fgfr2* murine embryos, implying compensation pathways or possible *Cre*-driver or timing-dependent differences.

##### 4.1.2.4 Pdgf (platelet-derived growth factor) signaling

Early evidence of Platelet-Derived Growth Factor (Pdgf) signaling in cardiac development emerged from the embryonic/neonatal lethality observed in germline deletion of *Pdgfa* or *Pdgfr*α in mice (Boström et al., 1996; Tallquist and Soriano, 2003). Lu et al. (2001) first discovered that Pdgf-BB induces EMT and SMC marker expression in quail, while Pdgf receptor stimulation similarly enhanced EMT in mice (Mellgren et al., 2008). Likewise, inhibition of Pdgf signaling impaired epicardial cell proliferation and coronary vasculature formation in zebrafish (Kim et al., 2010). In similar periods, Pdgfrβ expression in EPDCs was reported in quail-chick chimera (Guadix et al., 2006), as well as Pdgf-A expression in rat epicardial cell line and mouse epicardium and myocardium was confirmed (Kang et al., 2008). In the human fetal heart, Chong et al. (2013) demonstrated robust Pdgfrα expression in interstitial cells of the epicardium, myocardium, endocardium, and cVSMCs, with rare observation in endothelial cells and cardiomyocytes. However, significant Pdgfrα+ cardiomyocytes were observed in the adult diseased heart (Chong et al., 2013).

To assess tissue-specific roles of Pdgf signaling, Kang et al. (2008) deleted both Pdgf receptors (*Pdgfrα* and *Pdgfrβ*) in myocardium and mesoderm via *Mesp1* ^*Cre/+*^, but did not observe any myocardial or coronary vascular defects in mutants. However, Smith et al. (2011) deleted *Pdgfrα*, *Pdgfrβ*, or both in the mouse epicardium and observed significantly defective EMT. Interestingly, the loss of *Pdgfrα* resulted in a specific deficiency of cardiac fibroblasts, whereas *Pdgfrβ* deletion impaired the development of cVSMCs, highlighting distinct roles of epicardial Pdgfrα and Pdgfrβ in fate specification. Supporting the role of epicardial Pdgfrα in fibroblast formation, Rudat et al. (2013) further revealed that *Tbx18* ^*Cre/+*^ mediated loss of Pdgfrα prevents differentiation of EPDCs into mature fibroblasts. Interestingly, Ali et al. (2014) revealed strong Pdgfrα expression in endocardium-derived fibroblasts as well, expanding the understanding of Pdgfrα as a marker beyond epicardium-derived fibroblasts (pan fibroblast marker). This was further validated using the *collagen1a1-GFP* mouse model that labeled both epicardium- and endocardium-derived Pdgfrα+ fibroblasts (Moore-Morris et al., 2014).

Focusing on Pdgfrβ, Mellgren et al. (2008) revealed the absence of coronary vasculature and thinner myocardium in *Pdgfrβ−/−* mutant hearts. Interestingly, epicardium-specific deletion of Pdgfrβ showed region-specific defects in the coronary vasculature. This indicates that Pdgfrβ signaling from other sources, in addition to the epicardium, might contribute to the development of coronary vasculature. Further contribution of Pdgf signaling in coronary vasculature formation will be discussed in Section 4.4 later in this review.

Multiple downstream effectors have been reported to modulate Pdgf signaling. According to Mellgren et al. (2008), Pdgf receptor stimulation enhanced EMT via Pdgfrβ-PI3K pathway. A recent finding in zebrafish models (Shrestha et al., 2023) further revealed PI3K-Pdgfrα signaling to regulate latero-medial migration of cardiomyocytes in early heart tube formation. Additionally, Smith et al. (2011) demonstrated the Pdgfr-Sox9 axis regulating migration and cytoskeletal organization of epicardial cells. Furthermore, Guadix et al. (2011) introduced the epicardial Pdgf/Pdgfrα-RA axis in heart development. They observed low expression of Pdgfrα in *Wt1* null-embryos and immortalized epicardial cells, which were rescued by exogenous RA.

##### 4.1.2.5 Tgfβ signaling

Involvement of transforming growth factor β (Tgfβ) signaling in non-autonomous epicardial regulation of myocardium was also prevalent. Takahashi et al. (2014) delineated that epicardial block-driven chick myocardial thinning results from immature sarcomere formation (reduced Z-line spacing) and smaller cardiomyocyte size (increased cell density). These defects were linked to reduced p-Smad2, p-Erk, and lower expression of Tgfβ2 and Fgf2. These suggest that Tgfβ and Fgf signaling play an essential role in epicardium-regulated cardiomyocyte growth and sarcomere maturation. To note, Tgfβ signaling mostly correlates with epicardial EMT-based regulation of heart development and will be discussed in detail under 4.2.1 section later in this review.

##### 4.1.2.6 Transcription factors

###### 4.1.2.6.1 Wt1

Multiple transcription factors have been reported over time as influencers of non-autonomous epicardial roles. Following the striking phenotype of *Wt1* null embryos (Moore et al., 1999; Kreidberg et al., 1993) and confirmed myocardial invasion of Wt1+ EPDCs via genetic labeling (Zhou et al., 2008; Zhou et al., 2010), Martínez-Estrada et al. (2010) generated epicardium-specific *Wt1* KO, inducible *Wt1* KO epicardial cell line, and *Wt1* KO embryoid bodies (differentiated from *Wt1* null embryonic stem cells). All three systems showed reduced EMT markers and ectopic expression of epithelial markers in epicardial cells. They also implied that Wt1-regulated EMT might influence the formation of cardiomyocytes. Later, a study by von Gise et al. (2011) proved that *Wt1* KO hearts showed defective EPDCs migration and invasion in the myocardium, resulting in diminished proliferation of compact myocardium. Mechanistically, impaired expressions of canonical Wnt/β-catenin signaling components (Lef1 and β-catenin) and downstream targets (Axin2, Cyclin D1, and Cyclin D2), and activity of Wnt/β-catenin reporter transgene were observed in *Wt1* KO epicardial cells. Additionally, Wnt5a, a non-canonical Wnt, and Raldh2 were markedly downregulated, supporting findings by Guadix et al. (2011).

Furthermore, Wt1 represses chemokines *Ccl5* and *Cxcl10* (inhibitors of EPDC migration and cardiomyocyte proliferation) in epicardial cells by upregulating Irf7 (interferon 7) (Velecela et al., 2013; Sanchez-Fernandez et al., 2022), revealing interferon-dependent regulation of myocardial growth.

###### 4.1.2.6.2 NF-κB

A growing body of evidence highlights nuclear factor kappa B (NF-κB) as a central modulator of epicardial-myocardial communication. Early insight came from Craig et al. (2010a), who demonstrated that high molecular weight hyaluronan (HMW-HA) stimulates epicardial cell invasion and differentiation *in vitro*, via NF-κB signaling. According to Clark et al. (2016) and DeLaughter et al. (2016), NF-κB also regulates Tgfβ receptor 3 (Tgfβr3) mediated epicardial cell invasion. *Tgfbr3* ^*+/+*^ epicardial cells treated with NF-κB inhibitor failed to invade in response to a variety of pro-migratory ligands. Li Y. et al. (2017) performed 2D LC-MS/MS–based secretome profiling of chick epicardial–myocardial co-cultures and identified NF-κB as a top-predicted transcriptional regulator. Although NF-κB inhibition blocks EMT in both chick and mouse epicardial cells, the authors noted a species-specific difference in NF-κB localization: it was predominantly nuclear in chick epicardial cells but largely perinuclear in mice. Thus, a potential difference in NF-κB activation dynamics can be implied.

###### 4.1.2.6.3 Ets, Hif-1α, Tcf21, TFEB, Snai1b, Sox9

Expression of transcription factor Ets1 was observed in EMT regions during heart development (Macías et al., 1998). Lie-Venema et al. (2003) introduced an antisense *Ets* sequence via bloodstream in chick embryos. Despite this broad distribution, strong phenotypes such as reduced EMT, thinner mesenchyme, disrupted coronary vascular system with myocardial-to-subepicardial fistulae, and impaired myocardial expansion (thinner wall, broader and fewer trabeculae)—all implicated epicardial dysfunction as the primary source of the defects. However, in mammals, detailed cardiac analysis for Ets functioning is not available yet, possibly because of mid-gestational lethality in *Ets1* and *Ets2* null mice (Barton et al., 1998; Yamamoto et al., 1998; Lie-Venema et al., 2007).

Tao et al. (2013) revealed Hypoxia inducible factor-1α (Hif-1α) as another critical regulator. In their study, adenoviral delivery of constitutively active *Hif-1α* (*ca Hif1α*) in avian embryos caused enhanced EMT but marked defects in myocardial invasion of EPDCs. They revealed that Hif-1α–driven upregulation of Flt-1 impaired Flk-1 signaling required for proper myocardial invasion.

Boezio et al. (2023) revealed a temporal regulation of epicardial-myocardial crosstalk by using a temporally controlled tcf21:NTR/MTZ ablation system in zebrafish. While early loss of epicardial cells led to reduced CM proliferation, restoration of epicardial coverage at a late stage via MTZ washout successfully rescued myocardial growth. This suggests that epicardial influence might be dispensable once complete coverage is achieved in zebrafish. In addition to reduced *Fgf24* and *Vegfaa* expression, mitochondrial and ribosomal genes were significantly downregulated in mutant cardiomyocytes. This suggests that the epicardial defect may cause mitochondrial insufficiency and defects in CM maturation.

Astanina et al. (2022) uncovered transcription factor EB (TFEB) as an EMT suppressor. *TFEB* overexpression in the mouse epicardium caused severely impaired EMT and EPDC differentiation along with defective myocardial and interventricular septal thickening. Functionally, TFEB acts through TGIF1 to suppress Tgfβ/Smad-mediated EMT. Gentile et al. (2021) identified an additional role of transcription factor *Snai1b* beyond its traditional EMT function in zebrafish. *Snai1b*-deficient embryos showed cardiomyocyte extrusion into the pericardial cavity, especially in the regions under mechanical stress. These CMs showed increased contractility with weakened adhesion. Thus, *Snai1b* suppresses cardiac contraction to reduce extrusion and preserve CM integrity during heart development.

Most recently, epicardial deletion of transcription factor *Sox9* revealed impaired EPDCs invasion into the walls and AV valves, resulting in a thinner myocardial wall. Postnatally, mutants’ phenotype resembles human myxomatous mitral valve disease (MVD), characterized by extracellular matrix disorganization. Authors further identified a novel role of *Cd109*s association behind the observed valve pathology (Harvey et al., 2024).

#### 4.1.3 2016–2025: emergence of noncoding RNAs and epigenetic regulators

Epigenetic regulation of the epicardium-myocardium crosstalk has become the focus of recent explorations. Deletion of micro-RNA processing enzyme Dicer in proepicardium led to impaired EMT and reduced epicardial proliferation and differentiation into cVSMCs (Singh et al., 2011). Pontemezzo et al. (2021) revealed a novel microRNA mediated mechanism behind Tgfβ1-induced EMT in murine epicardial-mesothelial cells (EMC). Specifically, Tgfβ1 stimulation upregulated epicardium-derived cardiogenic factor Follistatin-like 1 (Fstl1) by suppressing its repressor miR-200c-3p. These findings establish a novel Fstl1-miR-200c-3p regulatory axis for epicardial behavior.

A novel post-transcriptional splicing mechanism emerged from a study by Jackson-Weaver et al. (2020), who identified Prmt1 as a splicing regulator of epicardial EMT and differentiation. Deletion of *Prmt1* in the mouse epicardium resulted in blocked EMT, impaired invasion, and reduced formation of EPDCs. Functionally, loss of *Prmt1* causes accumulation of p53, which suppresses EMT via enhanced Slug degradation. Prmt1 affects p53 expression by regulating alternative splicing of p53 regulator Mdm4, hence fine-tuning the Prmt1-Mdm4-p53-Slug axis. A deeper look into epigenetic repression was provided by Jang et al. (2022), who demonstrated that Hdac3 regulates epicardial mitogen expression via microRNA suppression. Conditional deletion of *Hdac3* in the epicardium led to significant thinning of the compact myocardial layer with unaffected trabeculae, reduced EPDCs, and EMT markers. Mechanistically, they identified that microRNA (miR)-322 and miR-503 were upregulated, repressing Fgf9 and Igf2 expression in *Hdac3* KO cells. This study uncovered a double-negative axis where *Hdac3* represses key mitogen suppressing miRNA, thus placing the epicardium as a non-cell-autonomous epigenetic regulator of compact myocardium expansion.

#### 4.1.4 Others

Beyond epigenetic regulators, several additional mediators have been identified that regulate epicardial behavior and, in turn, influence myocardial development. Mahtab et al. (2008) revealed that *podoplanin* KO mice show hypoplastic and perforated compact and septal myocardium and reduced EMT. Weeke-Klimp et al. (2010) explored how EPDCs influence cardiomyocyte development by co-culturing quail EPDCs with neonatal mouse cardiomyocytes. They revealed that only direct co-culture enhanced CM proliferation, maturation, and alignment. In contrast, indirect (using transwell inserts to separate cells while sharing medium) culture with EPDCs conditioned medium failed to induce these effects. However, their findings contrast with Takahashi et al. (2014), who rescued myocardial defects in chick using EPDCs conditioned media.

Another study by Iyer et al. (2016) observed that Loss of *Crim1* (transmembrane protein expressed in epicardium and EPDCs) function leads to epicardial defects and hypoplasia. Epicardium-restricted deletion of *Crim1* further resulted in reduced proliferation of EPDCs and differentiation into cardiac fibroblasts in mutants, which is mediated by phosphorylation of Smad2 and Erk1/2. At a similar time point, Arora et al. (2016) reported the role of angiogenic hormone, prokineticin-2 (Pk2) and its receptor Pkr1 as epicardial regulators (Olivey and Svensson, 2010). Pk2 induced EMT in epicardial cells, and *Pkr1* deletion in epicardium caused markedly diminished EPDC formation, reduced ventricular wall thickness with impaired proliferation, and disrupted coronary vessel development via PI3K/Akt pathway.

Ridge et al. (2017) investigated the role of non-muscle myosin heavy chain IIB (NMHC IIB), encoded by *Myh10*, in heart development using a mouse model possessing a splice-donor site mutation in *Myh10*. Mutants showed NF-κB-mediated disorganized, hyperproliferative epicardium, impaired EMT, thin ventricles, and septal defects. Junghof et al. (2022) recently identified type II classical cadherin Cdh18 as a novel biomarker for epicardial cells in a human induced pluripotent stem cell (hiPSC)-derived epicardial differentiation model (Junghof et al., 2022). They further identified that siRNA-mediated knockdown of Cdh18 led to a shift in differentiation towards SMCs, rendering Cdh18 essential for epicardial fate specification.

##### 4.1.4.1 Calcium signaling mediated epicardial regulation of heart development

Proper Calcium (Ca^+2^) homeostasis is essential for cardiac contraction, and epicardium/EPDCs can influence heart morphogenesis by interfering with Ca^+2^ signaling-regulated contractility. Weeke-Klimp et al. (2010) revealed that EPDCs can affect cardiomyocytes’ Ca^+2^ handling to regulate their alignment, maturation, and contraction. A key player in Ca^+2^ homeostasis in sarcoplasmic reticulum (SR) Ca^+2^ ATP ase (SERCA2a), which regulates Ca^+2^ mediated cardiac muscle contraction and relaxation by sequestration of cytosolic Ca^+2^ and uptake into SR. Co-culturing quail EPDCs with neonatal mice cardiomyocytes revealed increased expression of electrical and mechanical junction proteins as well as upregulated SERCA2 in cardiomyocytes, indicating enhanced Ca^2+^ handling and more mature sarcomeric organization. Aligned sarcomere patterns observed further correlated with improved cardiomyocyte organization. Similarly, Iyer et al. (2015) demonstrated that human pluripotent stem cell-derived epicardial cells can recapitulate epicardial morphology and marker expression. Moreover, these cells differentiate into SMCs upon ligand stimulation. Interestingly, these SMCs displayed contractility, evidenced by increased intracellular Ca^2+^ signaling when exposed to cholinergic receptor agonists. Kelder et al. (2015) further supported the epicardium’s role in regulating cardiac physiology, showing that the chick epicardium expresses β-adrenergic receptors and modulates heart rate, contraction, and conduction by responding to epinephrine. In their study, epicardium-blocked hearts exhibited significantly reduced epinephrine responses, leading to slower heart rates. Given the central role of Ca^2+^ in cardiac contraction, these results also imply dysregulated Ca^2+^ signaling under epicardial dysfunction, which could be a new area to be explored with this model.

Together, these studies highlight the critical roles of the epicardium/EPDCs in regulating cardiomyocyte contractility and alignment via Ca^2+^ signaling, thereby influencing heart development and function.

A comprehensive summary of key experimental models and associated signaling that advances our understanding of epicardial-myocardial crosstalk has been provided in Table 3.

**TABLE 3:** A comprehensive summary of key experimental models and associated signaling involved in epicardium-myocardium crosstalk during development.

Study (Year)	Model/Lethality	Gene-pathway/Methodology	Key findings	Interpretation
Männer (1993)	Chick/∼7% death	PE ablation microsurgery	Epicardium and myocardial defects	Epicardium is derived from pericardial epithelium and epicardium might be essential for myocardial growth
Kwee et al. (1995), Yang et al. (1995)	Mouse/Before E11.5-E12.5	Global deletion of VCAM-1, α4-integrin	No epicardium, thin myocardium, septal defects and severe coronary dysfunction	Implication for epicardial regulation of heart growth
Moore et al. (1999)	Mouse	Wt1/Global KO	Lack of definitive epicardium, myocardial hypoplasia	WT1 is present in epicardium, and critical for epicardium development and possible myocardium
Morabito et al. (2001)	Chick	Primary explants/Test for EMT regulator/Collagen gel EMT assay	Fgf2 induces EMT; Tgfβ-1/2/3 antagonizes	Found key Regulators of EMT
Dettman et al. (2003)	Chick	α4-integrin/adenoviral downregulation in explant	Enhanced EMT and premature vascularization	α4-Integrins prevent premature EMT
Stuckmann et al. (2003)	Chick	Heart slice culture	Epicardial RA/EPO induce epicardial mitogen release	Epicardial relay of systemic signals
Pennisi et al. (2003)	Chick	Epicardium loss/PE ablation	↓Fgfr1/2 levels but preserved patterning	Epicardial is not required for transmural pattern maintenance, but required only for mitogen expression level
Merki et al. (2005)	Mouse/36.5% survive upto postnatal	RXRα/epicardial KO	Myocardial and coronary defect: ↓Fgf2, Wnt9b, failed EMT, coronary defects	RA-Fgf-Wnt feedback in epicardial signaling
Lavine et al. (2005)	Mouse	Fgf9, 16, 20/Fgfr1/2: Conditional deletion	Endo/epi FGF → Fgfr1/2 → myocardial proliferation	Ligand redundancy and epicardial autonomy
Kim et al. (2010)	Zebrafish	Induction of Pdgf signaling/Primary explant	↓Pdgf→ ↓EMT, ↓mural markers	Pdgf signaling regulates epicardial EMT and mural differentiation
Torlopp et al. (2010)	Chick	Exogenous Fgf mediated stimulation/PE explant	↑Fgf2 → ↑ MAPK/PI3K→PE ↑EMT/proliferation	Autocrine/paracrine FGF maintains PE growth and EMT capacity
Craig et al. (2010a)	Chick	HMW-HA stimulation of epicardium/*in vitro*	HMW-HA↑→NF-κB activation↑	NF-κB integrates ECM-derived signals for epicardial EMT
Brade et al. (2011)	Mouse	Raldh2, Rxra/Global deletion	Liver RA → EPO → Igf2 in epicardium	Extracardiac regulation of epicardial Igf signaling influences myocardium growth
Smith et al. (2011)	Mouse	Pdgfrα/Pdgfrαβ/Epicardial KO	Pdgfrα → fibroblast; Pdgfrβ → cVSMC	Lineage-specific EMT via Pdgf signaling
Vega-Hernández et al. (2011)	Mouse	Fgf10/Fgfr2b/conditional deletion	Myocardial Fgf10 → EPDC migration, wall growth	Impaired EDPC invasion affects myocardium via Fgf pathway
Tao et al. (2013)	Avian	HIF-1α, Vegf signaling/(adenoviral caHIF-1α delivery)	↑HIF-1α →↑EMT and vascular SMC differentiation but blocks myocardial invasion via upregulation of Vegf decoy	Hypoxia signaling in epicardium regulates EPDC invasion through Vegf pathway
Trembley et al. (2015)	Mouse/50% neonatal lethality	Myocardin transcription factor Mrtfa and Mrtfb/epicardial deletion	Diminished cell migration and coronary defect	Mrtfa and Mrtfb regulate migration and invasion in Tgfβ dependent pathway
Shen et al. (2015)	Mouse	Igf2/Mesoderm, epicardium and endocardium specific deletion	Early phase: liver RA → EPO → epicardial EPOR → Igf2; late phase: placental glucose and oxygen	Biphasic regulation of epicardial Igf2 to regulate heart growth
Arora et al. (2016)	Mouse/Partial embryonic lethality (∼15% in Wt1^*Cre/+*^)	Pkr1/epicardial-specific deletion	Pkr11↓→ PI3K/Akt↓→EMT, reduced EPDC formation↓, ventricular walls↓, coronary vasculature↓	Epicardial Pkr1 signaling via PI3K/Akt is crucial for EMT, coronary vessel development, and myocardial growth
Clark et al. (2016)	Mouse	Role of TgfβR3 in epicardial cell invasion/epicardial cell culture	TgfβR3-T841A mutant form functions ligand independently. NF-κB inhibition blocked ligand dependent and independent invasion	NF-κB is a convergent node downstream of diverse EMT-stimulating signals
Iyer et al. (2016)	Mouse	Crim1 transmembrane protein/global and epicardium-restricted KO	Crim11↓→ Smad2/phospho-Erk1/2↓→EMT and proliferation↓, reduced fibroblast formation↓, ventricular walls↓	Crim1 modulates epicardial EMT and EPDC proliferation to impact myocardial development
Li et al. (2017b)	Chick	Identification of EMT Effector/Epicardial-myocardial Co-culture	Tgfβ2/PdgfBB → NF-κB → EMT; inhibition blocks EMT	NF-κB as EMT effector in epicardium
Yan et al. (2018)	Mouse primary epicardial cell culture	Igfr1 and ligand	Igfr1 expressed in epicardium E11.5–E17.5; regulates epicardial proliferation via FAK pathway	Igf–FAK axis supports epicardial cell proliferation, indirectly influencing myocardium
Jackson-Weaver et al. (2020)	Mouse	Prmt1/Epicardial KO	Stabilizes p53 → ↓Slug → blocked EMT	Prmt1–p53–Slug axis controls EMT
Dueñas et al. (2020)	Chick	miR-195/Overexpression in PEO	Promotes CM fate via Smurf1/Foxp1	MicroRNA controls epicardial fate
Dronkers et al. (2021)	Human	Fetal and adult Primary epicardial cell isolation method	Activin A and its receptor ALK4 are novel regulators of epicardial plasticity	Provides a human EPDC culture system to dissect epicardial EMT regulation and signaling pathways
Pontemezzo et al. (2021)	Murine epicardial-mesothelial cells (EMCs) *in vitro*	miR-200c-3p and FSTL1	miR-200c-3p targets Fstl1 to control EMT	Epicardial EMT is post-transcriptionally regulated by miRNAs targeting cardiogenic factors like FSTL1
Junghof et al. (2022)	hiPSC-derived epicardial cells	Cdh18 (classical cadherin)	Cdh18 marks epicardial lineage; loss triggers EMT and differentiation towards cardiac SMCs	Cdh18 is a key biomarker and regulator of EMT and lineage specification in humans
Jang et al. (2022)	Mouse	Hdac3 → Igf signaling	Epicardial HDAC3 represses miR-322 and miR-503 that suppress mitogens Igf2 and Fgf9 to control myocardial proliferation	HDAC3 epigenetically regulates epicardial IGF signaling
Astanina et al. (2022)	Mouse/Embryonic lethality by E15.5 (overexpression)	TFEB epicardial overexpression and knockdown)	↑TFEB→ ↓EMT via TGIF1-mediated repression of Tgfβ signaling	TFEB acts as EMT suppressor
Meier et al. (2023)	Human epicardioids (hPSC-derived 3D model)	Igf2/Igf1R and Nrp2 signaling	hPSC-derived epicardioids mimic human epicardial-myocardial signaling; RA-dependent patterning; Igf2/Igf1R and Nrp2 signaling	Development of Epicardioid helps to decipher human epicardial cells biology
Jiang et al. (2023)	Mouse/E12.5 and E13.5	Ezh2/Epicardial deletion	Impaired epicardial EMT, myocardial hypoplasia ↑TIMP3 upregulation mediated extracellular matrix reconstruction	Epicardial Ezh2 controls ECM-mediated myocardial growth
Boezio et al. (2023)	Zebrafish	tcf21−/−, wt1a−/−, MTZ ablation	↓CM size, ↓Fgf/Vegfaa, early epicardial window	Early, not late-stage defect in epicardial coverage cause myocardium defect
Wang et al. (2024)	Mouse/Midgestational lethality	Ccm2/Epicardial cKO	Polarity/cytoskeletal defects, altered ECM, LV noncompaction	Ccm2 mediated EPDC adhesion and migration regulate myocardium formation

### 4.2 Cell-autonomous regulation of epicardium

Several aspects of epicardial regulation, such as polarity, cell division, proliferation, survival, and EMT, shape cardiac development by ensuring proper epicardial coverage. Wu et al. (2010) revealed the requirement of a specific orientation of cell division: perpendicular division to undergo EMT. β-catenin/Numb-N-cadherin complex regulates EMT by maintaining spindle orientation, cell polarity, and directed cell division. Wu et al. (2013) revealed defects in epicardial cell’s proliferation and coronary vessel formation in *Tbx18* null mice models, associated with alterations in several components of Hedgehog and Vegf signaling. However, Greulich et al. (2012) did not observe obvious defects in epicardial EMT and differentiation by either loss of *Tbx18* function or prolonged functioning of *Tbx18*. Rather, epicardial mis-expression of a transcriptionally active form *Tbx18VP16* resulted in premature differentiation to SMCs. Supporting this, Wu et al. (2013) revealed Tbx18 as a SRF/CArG box dependent repressor of SMCs differentiation, evident by reduced SMCs marker in *Tbx18* overexpressed cells *in vitro*. Interestingly, Takeichi et al. (2013) demonstrated that Tbx18 and Wt1 bi-directionally regulate the epicardial EMT by affecting Slug expression in murine primary epicardial cells. Knockdown of *Wt1* induced the epicardial EMT, while knockdown of *Tbx18* inhibited the *Wt1* and Tgfβ1-induced mesenchymal transition. This dynamic regulation suggests that fine-tuning of Tbx18-Wt1 activity is essential to ensure proper EMT.

Beyond transcriptional regulation, Baek and Tallquist (2012) identified another EMT regulator, Neurofibromin (Nf-1), a Ras-GTPase activating protein (GAP), mutation of which is traditionally connected to tumorigenesis. Specific deletion of *Nf-1* in epicardium enhanced EMT and increased the cVSMCs and fibroblasts in myocardium in the Pdgf-dependent pathway. Similarly, Li J. et al. (2017) revealed another small GTPase, Cdc42, as a crucial regulator of PEO translocation. *CDC42* conditional deletion disrupted the formation of villous projections and cysts that carry PEO cells to the myocardium, leading to incomplete epicardial coverage.

#### 4.2.1 Tgfβ signaling mediated epicardial EMT

Many studies have dissected the complex, often context-dependent role of Tgfβ signaling in epicardial EMT and differentiation. Initial evidence by Morabito et al. (2001) revealed that Tgfβ1 weakly stimulates EMT in chick epicardial explants, while Tgfβ2/3 showed no EMT induction, instead, inhibited Fgf2-induced EMT. In contrast, Compton et al. (2006) and Austin et al. (2008) identified Tgfβ1/2/3 as a strong promoter of EMT and smooth muscle differentiation in both chick and mouse epicardial models, in which Tgfβ receptor 1 (Alk5) and downstream RhoA/p38 MAPK play as key effectors. Sánchez and Barnett (2012) further revealed that Tgfβ and Bmp2-induced EMT mediates through Alk5 and identified a different Par6–Smurf1–RhoA regulatory axis. Genetic studies in mice by Sridurongrit et al. (2008) demonstrated that epicardial—but not myocardial or endocardial—deletion of *Alk5* impaired EMT, smooth muscle formation, and myocardial growth, indicating a cell-autonomous requirement of Alk5 in the epicardium (Dronkers et al., 2020). Meanwhile, Olivey et al. (2006) listed another type 1 Tgfβ receptor isoform (Alk2) for early-stage epicardial EMT regulation, functioning through the Smad pathway.

Interestingly, Sánchez et al. (2011) discovered that GIPC1-binding sites on the Tgfbr3 cytoplasmic tail are indispensable for ligand interaction, followed by EMT and invasion. Supportively, Hill et al. (2012) highlighted distinction in ligand-receptor interaction: Bmp2 binds with Alk3 to induce epithelial loss but not SMC differentiation, while Tgfβ requires Alk5 and supports both EMT and SMC fate. Both require Tgfβr3–GIPC1 interaction, again underscoring the importance of cytoplasmic domain signaling.

A pivotal discovery by Clark et al. (2016) and DeLaughter et al. (2016) established NF-κB as a shared, essential effector downstream of Tgfβr3. A specific *T841A-TGFβR3* mutant variant, possibly resistant to internalization, enabled ligand-independent but still NF-κB-dependent EMT induction, suggesting parallel and converging Alk–NF-κB axes. Additionally, Dronkers et al. (2021) uncovered Activin A–Alk4 signaling as an independent epicardial EMT pathway. Adding to this, Lupu et al. (2020) revealed that Alk4 was highly expressed in fetal epicardial cells (Moerkamp et al., 2016), and overexpressing Alk4 in adult epicardial cells mimicked fetal epiMT, highlighting a role of Alk4 in the developmental switch.

Interestingly, Tgfβ-neutralizing antibodies could not block spontaneous EMT in fetal EPDCs, but the combination with Activin A inhibitor blocked, implying compensatory interplay between Activin A and Tgfβ. Their work also addressed a methodological issue: SB431542, which is widely used in myriads of previous studies as a Tgfβ/Alk5 inhibitor, also blocks ALK4/7, thus potentially could overlook activin’s contributions in Tgfβ signaling in previous studies.

Moerkamp et al. (2016) introduced a novel human fetal and adult EPDCs isolation and culture method. Using this method, they characterized that Alk5 inhibition is essential to retain the epithelial phenotype and inhibit spontaneous EMT of fetal EPDCs, making it a tool to study epicardial state transitions. Interestingly, adult EPDCs showed Tgfβ stimulated invasion and formation of tube-like structures, while fetal EPDCs reduced their migration upon Tgfβ stimulation and failed to show tube-like structures, pinpointing potentially different mechanisms of Tgfβ responsiveness.

### 4.3 Epicardium-derived cardiac fibroblasts influences myocardial growth

A subset of EPDCs differentiates into interstitial fibroblasts and plays critical roles in myocardial growth and organization. Vega-Hernández et al. (2011) discovered that interference of Fgf signaling in murine hearts resulted in a deficiency in epicardium-derived cardiac fibroblasts in the myocardium, leading to reduced cardiomyocyte proliferation. This highlights that the presence of EPDCs underlying the myocardium is crucial for myocardial growth.

Expanding on this, Barnes et al. (2011) revealed that deletion of transcription factors *Hand2* in *Hand1*-or *Wt1*-expressing cells caused epicardial disorganization, non-compaction and outflow tract defects. Mutants had a deficiency in fibroblasts because of lineage shifting from Pdgfrα^+^ (fibroblast-biased) to Pdgfrβ^+^ (vascular-biased) EPDCs. They further identified that a disrupted balance between fibronectin and its receptor disrupts proper ECM assembly, which is critical for fibroblast to anchor in the myocardium, thereby causing myocardial compaction defects, as reviewed in George and Firulli (2019). Also, ablation of cardiac fibroblast mediated by *Pdgfr*α*-CreER* controlled DTA system caused myocardial and vasculature defects in developing heart (Deng et al., 2025).

Recently, Xiao et al. (2018) demonstrated that deletion of Hippo signaling kinases *Lats1/2* in mice epicardium manifests a significant deficiency in EPDCs differentiated cardiac fibroblasts. Instead of achieving a fibroblast fate, EPDCs remained in a transitional state, co-expressing both epicardial and fibroblast markers. Mechanistically, the increased nuclear localization of Yap interfered with fibroblast differentiation by disrupting RA synthesis and extracellular matrix remodeling. Supporting this finding, cells grown on stiffer substrates exhibited more nuclear Yap as well as reduced fibroblast marker expression. These findings suggest that mechanical environments can modulate Hippo signaling to affect epicardial cell fate and myocardial growth. Further involvement of Hippo pathway has been discussed in Section 4.4.

### 4.4 Epicardium regulated coronary vasculature formation influences heart development

Epicardial signaling also exerts essential control over coronary vasculature formation of the heart during development. For example, epicardial *Gata4/6* deletion affected endothelial cell recruitment and plexus development, indicating epicardial necessity in vascular formation (Kolander et al., 2014). Multiple downstream pathways direct EPDCs differentiation into cVSMCs. Landerholm et al. (1999) showed that Serum Response Factor (SRF) is indispensable for cVSMCs differentiation but not for EMT. Similarly, RhoA signaling promotes cVSMCs development by supporting EPDC survival and migration (Lu et al., 2001). Epicardial Wnt/β-catenin signaling, as illustrated by Zamora et al. (2007), is dispensable for epicardial formation, but essential specifically for EPDC invasion and SMCs differentiation. Furthermore, as studied by Smart et al. (2007), Thymosin β4 (Tβ4) was shown to be both necessary for EPDC migration and differentiation into coronary vascular lineages. Loss of *Tβ4* in the heart leads to the entrapment of Tie2^+^ endothelial and α-actin^+^ smooth muscle cells in the epicardium, leading to defective coronary vasculature.

Environmental conditions also regulate epicardial signaling. Embryonic hypoxia impaired coronary development as well as compact myocardium formation (Nanka et al., 2008). Hypoxia promotes EMT and causes premature differentiation into cVSMCs through HIF-1α–Snail signaling, as delineated by Jing et al. (2016). Tao et al. (2018) further linked hypoxia to non-canonical Tgfβ-RhoA signaling, showing its necessity for EMT and SMC marker expression.

Additionally, Pdgfrβ signaling plays a critical role in cVSMCs formation. Mellgren et al. (2008) revealed that both epicardial and non-epicardial Pdgfrβ signaling are crucial for cVSMCs formation. Another major signaling pathway, Notch, functions in multiple axes to regulate epicardium. Grieskamp et al. (2011) showed that epicardial deletion of *Rbpj* (transcriptional regulator of Notch) disrupted EPDC differentiation into SMCs while differentiation into fibroblasts remained unaffected. del Monte et al. (2011) further dissected Notch-specific roles: epicardial *Notch1* deletion impaired coronary arteries and myocardial compaction via Raldh2 downregulation, while Notch2/3 were enriched in perivascular regions and drove SMCs maturation. These findings show spatially distinct roles for Notch in coordinating myocardial growth and coronary differentiation.

Myocardin-related transcription factors (MRTF) Mrtfa/b were shown by Trembley et al. (2015) to orchestrate EPDC motility and pericyte formation. Deletion of *Mrtfa/b* impaired EPDCs migration, causing subepicardial hemorrhage and pericyte loss (Quijada et al., 2020). Hippo signaling also intersects with vascular formation. Singh et al. (2016) found that *Sema3D* ^*Cre/+*^ mediated epicardial deletion of Yap/Taz disrupted EMT and coronary formation.

Cilia-related signaling, specifically Wdpcp, also modulates epicardial responses. Liu et al. (2018) showed *Wdpcp* mutant hearts had premature subepicardial plexus formation and reduced SMC coverage. They found cilia-mediated Shh responsiveness as a regulator of coronary remodeling. Recently, Palmquist-Gomes et al. (2023) demonstrated that *Itga4* deletion causes disrupted epicardial formation, leading to myocardial discontinuities and endocardial extrusion, which resembles congenital coronary artery fistula (CAF) morphology. Assessment of CAF structure with lineage tracing revealed epicardial origin of the outer smooth muscle walls and endocardial origin of the inner lining. This highlights the consequences of premature epicardial-myocardial contact and reinforces the structural role of epicardial adhesion in cardiac integrity.

### 4.5 Epicardium regulates myocardium via extracellular matrix (ECM) modulation

Multiple reports underscored the role of ECM components mediating epicardial regulation of myocardium in recent years (Bowers et al., 2022). Craig et al. (2010a) revealed that hyaluronan HMW-HA stimulates EPDCs differentiation and invasion into the myocardium, which is regulated by MEKK1 phosphorylation. Allison et al. (2015) identified that HA-mediated EPDCs invasion is regulated through Tgfbr3, further dependent on the downstream Src-RhoA/Rac1pathway. Craig et al. (2010b) further demonstrated that Tgfβ2 stimulates epicardial motility in mouse and human epicardial explants by indirectly enhancing *hyaluronan synthase 2* (*Has2*) expression and HA. Likewise, Sun et al. (2022) revealed that targeted ablation of hapln1a+/Tcf21+ EPDCs impairs HA deposition, reduces cardiomyocyte proliferation, and leads to a significantly thinner and disorganized compact layer in zebrafish (Foglio et al., 2024).

Matrix degradation has also been correlated with epicardial invasion. Combs et al. (2011) revealed that Nfatc1 regulates matrix degradation via Ctsk. Loss of *Nfatc1* impaired ECM breakdown, reducing EPDC invasion into the myocardium. Similarly, a recent study by Jiang et al. (2023) showed that epicardial Ezh2 is essential for matrix degradation during invasion. Ezh2 was found in human and mouse epicardium, and it acts as a repressor of TIMP3 (tissue inhibitor of metalloproteinase 3) to promote matrix degradation and epicardial cell migration. *Wt1* ^*Cre/+*^ mediated epicardial deletion of *Ezh2* showed impaired EMT associated with defective basement membrane degradation and myocardial hypoplasia.

Additional ECM regulators have been reported. According to Sun et al. (2021), basement membrane-associated proteoglycan agrin plays as a crucial regulator of epicardial EMT. Deletion of *agrin* caused impaired EMT, disruption in basement membrane integrity, and a reduction in Wt1+ EPDCs in myocardium. Functionally, agrin decreases β-catenin and promotes pFAK localization at focal adhesions to enhance EMT, according to findings from human embryonic stem cell-derived epicardial-like cells. Similarly, Bonet et al. (2022) reported that *Ccbe1* (ECM protein) KO mouse manifested thinner and hyper-trabeculated ventricular myocardium with reduced cardiomyocyte and epicardial cells proliferation.

At an earlier developmental stage, Nahirney et al. (2003) identified an extracellular matrix bridge (ECMB) in the pericardial cavity, which enables migration of PEO cells to the myocardium. One of the components of ECMB is heparan sulfate, degradation of which resulted in aberrant development of the chick primordial epicardium. This underscores the requirement of ECM components for proper transfer of PEO cells to the myocardium (Nahirney et al., 2003).

**FIGURE 1:**
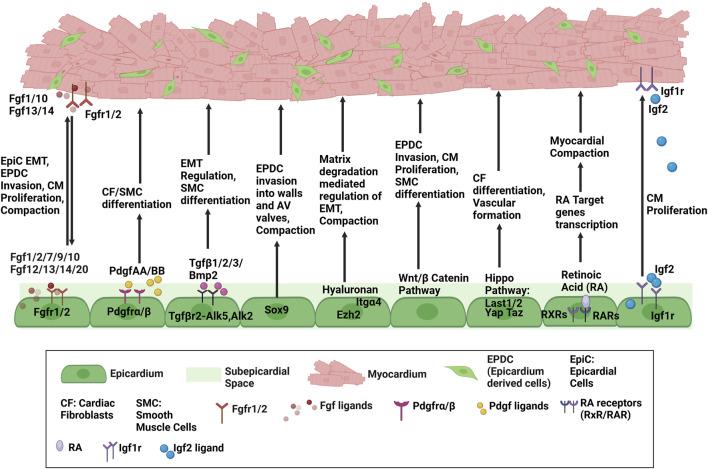
Schematic diagram highlighting major pathways regulating Epicardium-Myocardium Crosstalk.

Most recently, Wang et al. (2024) revealed Ccm2, a novel signaling molecule to regulate epicardium mediated heart development. Ccm2 is traditionally known for its association with cerebral cavernous malformations, and its epicardial-specific deletion resulted in myocardial thinning and early lethality. Functionally, *Ccm2*-deficient epicardial cells lose polarity and cell shape, exhibit Golgi mis-localization and disorganized actin stress with altered matrix and cytoskeletal genes. Thus, it highlights the requirement of cytoskeletal integrity and adhesion of EPDCs to myocardium for proper heart development. A comprehensive schematic diagram highlighting major pathways involved in epicardium myocardium crosstalk during development have been provided in Figure 1.

## 5 Conclusion and future prospects

In summary, the epicardium undergoes complex and dynamic interactions with the myocardium to regulate myocardial proliferation, growth and wall formation, EPDCs invasion, fibroblast differentiation, coronary vasculature formation, and extracellular matrix remodeling. Recent advances have heavily focused on unraveling the molecular complexity underlying these processes, yet myriads of fundamental aspects of epicardium-myocardium crosstalk remain unresolved and need attention to elucidate. For example, the lineage potential, molecular heterogeneity, and spatiotemporal dynamics of the epicardium remain an active area of controversy.

To fully understand the sophistication of epicardial regulation of heart development, a clear understanding of epicardial lineage contribution to the heart is necessary. As discussed throughout this review, several studies have reported epicardial contributions to cardiomyocytes and endothelial cells under certain experimental conditions; however, many of these conclusions came from *in vitro* or *ex vivo* systems. Also, recent studies reveal that even a trivial shift in intrinsic signaling or external cues can shift epicardial lineage fate. Therefore, precise control of experimental variables is required to avoid a misleading interpretation from epicardial lineage tracing.

Proepicardial and epicardial cell’s molecular heterogeneity is another complex field which is sparsely investigated. Weinberger et al. (2020) in zebrafish identified three distinct epicardial subpopulations, each with unique gene expression profile and role in cardiac morphogenesis. Dong et al. (2018) uncovered epithelial-mesenchymal hybrid state in many organs, including the heart, during mouse organogenesis via single-cell RNA sequencing (scRNA-seq). Recently, Farah et al. (2024) discovered several sub populations of EPDCs/cardiac fibroblasts in the ventricle with different expression profiles. Therefore, extensive high-resolution molecular profiling, such as scRNA-seq and ATAC-seq of mammalian models, is required to advance the understanding of the molecular and epigenetic heterogeneity within epicardial/EPDCs populations.

Spatiotemporal analysis of EPDCs populating the heart chambers is essential as epicardial contributions may vary between right and left ventricles during development, as suggested by Vicente-Steijn et al. (2015). They observed spatiotemporally distinct distribution of Wt1+ and Tcf21+ EPDCs between the two ventricles. Understanding these chamber-specific differences will provide insight into ventricle-specific differences in cardiomyopathies.

Another pressing knowledge gap is how epicardial dysfunction contributes to ventricular non-compaction cardiomyopathy (LVNC). A recent study Farah et al. (2024) has highlighted the necessity of Sema-Plexin signaling to mediate communication between compact zone fibroblasts with trabecular cardiomyocytes, during ventricular compaction. This highlights the breadth of unexplored areas of EPDCs and cardiomyocyte signaling to mediate the compaction process. Furthermore, epicardial-myocardial crosstalk likely involves intricate cellular communication that is still poorly explored. While long-distance endocardial cell-cardiomyocytes communication during development was intensively studied Miao et al. (2025), parallel studies exploring the bidirectional communication between epicardial cells/EPDCs and cardiomyocytes are urgently needed.

Translational research in epicardial biology is very promising yet remains at a very primary stage. Human stem cell-derived epicardial-like cells and epicardioids have emerged as valuable tools for studying epicardial behavior (Meier et al., 2023; Moerkamp et al., 2016; Sun et al., 2021). However, the conclusions from these models still need to be validated because of their imperfect recapitulation of the *in vivo* settings. Improving 3D organoid models, co-culture systems with cardiomyocytes, and testing with biomechanical factors are essential to refine these systems and advance human epicardial development and disease.

Finally, epicardial biology remains an underexplored therapeutic target in congenital heart disease and cardiomyopathies. A deeper understanding of epicardial heterogeneity, cellular and molecular level dynamics of EPDC-cardiomyocyte signaling may open new avenues for developmental studies and congenital heart disease research.
